# Modernizing persistence–bioaccumulation–toxicity (PBT) assessment with high throughput animal-free methods

**DOI:** 10.1007/s00204-023-03485-5

**Published:** 2023-03-23

**Authors:** Beate I. Escher, Rolf Altenburger, Matthias Blüher, John K. Colbourne, Ralf Ebinghaus, Peter Fantke, Michaela Hein, Wolfgang Köck, Klaus Kümmerer, Sina Leipold, Xiaojing Li, Martin Scheringer, Stefan Scholz, Michael Schloter, Pia-Johanna Schweizer, Tamara Tal, Igor Tetko, Claudia Traidl-Hoffmann, Lukas Y. Wick, Kathrin Fenner

**Affiliations:** 1 Helmholtz Centre for Environmental Research–UFZ, Permoserstr. 15, E04318 Leipzig, Germany; 2 Environmental Toxicology, Department of Geosciences, Eberhard Karls University Tübingen, Schnarrenbergstr. 94-96, E72076 Tübingen, Germany; 3 Helmholtz Institute for Metabolic, Obesity and Vascular Research (HI-MAG) of the Helmholtz Munich-German Research Centre for Environmental Health (GmbH) at the University of Leipzig and University Hospital Leipzig, Leipzig, Germany; 4 Environmental Genomics Group, School of Biosciences, University of Birmingham, Birmingham, B15 2TT UK; 5 Institute of Coastal Environmental Chemistry, Helmholtz Zentrum Hereon, Max-Planck-Straße 1, 21502 Geesthacht, Germany; 6 Quantitative Sustainability Assessment, Department of Environmental and Resource Engineering, Technical University of Denmark, Produktionstorvet 424, 2800 Kgs. Lyngby, Denmark; 7 Institute of Sustainable and Environmental Chemistry, Leuphana University Lüneburg, Universitätsallee 1, 21335 Lüneburg, Germany; 8 Institute of Biogeochemistry and Pollutant Dynamics, ETH Zürich, 8092 Zurich, Switzerland; 9 Comparative Microbiome Analysis, Environmental Health Centre, Helmholtz Munich - German Research Centre for Environmental Health (GmbH), Ingolstädter Landstr. 1, 85764 Neuherberg, Germany; 10 Research Institute for Sustainability–Helmholtz Centre Potsdam, Berliner Strasse 130, 14467 Potsdam, Germany; 11 Institute of Structural Biology, Molecular Targets and Therapeutics Centre, Helmholtz Munich - German Research Centre for Environmental Health (GmbH), Ingolstädter Landstr. 1, 85764 Neuherberg, Germany; 12 Environmental Medicine Faculty of Medicine, University of Augsburg, Stenglinstrasse 2, 86156 Augsburg, Germany; 13 Institute of Environmental Medicine, Environmental Health Centre, Helmholtz Munich - German Research Centre for Environmental Health (GmbH), Ingolstädter Landstr. 1, 85764 Neuherberg, Germany; 14 Department of Environmental Chemistry, Swiss Federal Institute of Aquatic Science and Technology (Eawag), 8600 Dübendorf, Switzerland; 15 Department of Chemistry, University of Zürich, 8057 Zurich, Switzerland; 16 International Sustainable Chemistry Collaboration Centre (ISC3), Friedrich-Ebert-Allee 32 + 36, D-53113 Bonn, Germany; 17 Department for Political Science, Friedrich-Schiller-University Jena, Bachstr. 18k, 07743 Jena, Germany

**Keywords:** Hazard assessment, New approach methodologies (NAMs), Persistence, Mobility, Biodegradation, In vitro bioassay, Toxicity

## Abstract

The assessment of persistence (P), bioaccumulation (B), and toxicity (T) of a chemical is a crucial first step at ensuring chemical safety and is a cornerstone of the European Union’s chemicals regulation REACH (Registration, Evaluation, Authorization, and Restriction of Chemicals). Existing methods for PBT assessment are overly complex and cumbersome, have produced incorrect conclusions, and rely heavily on animal-intensive testing. We explore how new-approach methodologies (NAMs) can overcome the limitations of current PBT assessment. We propose two innovative hazard indicators, termed cumulative toxicity equivalents (CTE) and persistent toxicity equivalents (PTE). Together they are intended to replace existing PBT indicators and can also accommodate the emerging concept of PMT (where M stands for mobility). The proposed “toxicity equivalents” can be measured with high throughput in vitro bioassays. CTE refers to the toxic effects measured directly in any given sample, including single chemicals, substitution products, or mixtures. PTE is the equivalent measure of cumulative toxicity equivalents measured after simulated environmental degradation of the sample. With an appropriate panel of animal-free or alternative in vitro bioassays, CTE and PTE comprise key environmental and human health hazard indicators. CTE and PTE do not require analytical identification of transformation products and mixture components but instead prompt two key questions: is the chemical or mixture toxic, and is this toxicity persistent or can it be attenuated by environmental degradation? Taken together, the proposed hazard indicators CTE and PTE have the potential to integrate P, B/M and T assessment into one high-throughput experimental workflow that sidesteps the need for analytical measurements and will support the Chemicals Strategy for Sustainability of the European Union.

## Introduction

Production of chemicals has doubled since 2000 and is increasingly endangering human health and the environment (Persson et al. 2022). Chemical risk assessment and risk management have not kept pace with the growing number and structural diversity of organic chemicals used in commerce (Kosnik et al. 2022). Synthetic organic chemicals are polluting the outdoor and indoor environment thereby threatening biodiversity (Hallmann et al. 2017) and increasing the prevalence of diseases (Landrigan et al. 2018; Neel and Sargis 2011; UNEP 2019), particularly non-communicable diseases and allergies (Celebi Sozener et al. 2022). Recent reports posit that the safe operating space for chemicals in the environment has been exceeded (Persson et al. 2022).

While society aims for a circular economy, it neglects the dangers posed by chemical pollution (Kümmerer et al. 2020). Within the contemporary discussion on establishing a circular economy, the topics of elemental flows of carbon, nitrogen and phosphorus are often prioritized over the fate of synthetic organic chemicals (e.g., pharmaceuticals, pesticides, consumer product chemicals), which are either ignored or only marginally considered as “novel entities”. This is a grave oversight, as chemical safety can be jeopardized in a circular economy by, for example, the accumulation of toxic chemicals in recycled products (Fantke and Illner 2019; Lowe et al. 2021) or the build-up of persistent aquatic pollutants during water recycling and reuse, which has been recognized in the regulation on minimal requirements for water reuse (EP&EC 2020).

The current risk assessment paradigm dates back to the early 1970 and includes an initial hazard assessment, followed by parallel exposure and effect assessment, ultimately bringing those elements together in a risk characterization (enHealth 2004; EP&EC 2006; NRC 1983; US. EPA 1976). Regrettably, these legacy risk assessment practices are slow, expensive, rely on animal testing, and are constantly outpaced by chemical innovation. Regulators were unprepared for the sheer numbers of new chemicals to assess, including the potential cumulative toxic action of chemicals in mixtures and the ability to discriminate subtle effects that chemical exposure can have on the environment and human health (Fenner and Scheringer 2021).

The European Union’s (EU) regulation REACH, which stands for Registration, Evaluation, Authorization and Restriction of Chemicals (EP&EC 2006), has improved this situation by implementing the principle “no data – no market” by placing the burden of chemical safety assessment onto the industry. The assessment of persistence, bioaccumulation, and toxicity (PBT assessment) was introduced into REACH as a new element of hazard assessment to identify “substances of very high concern” (SVHC). SVHC are chemicals that are classified as PBT or very persistent, very bioaccumulative (vPvB) or pose specific health hazards by being carcinogenic, mutagenic or reproductively toxic (CMR) or generate equivalent concern, such as chemicals that contain endocrine-disruptive properties and/or cause neurotoxicity. It has been also proposed that persistent, mobile and toxic (PMT) chemicals should trigger an equivalent level of concern as PBT chemicals (Hale et al. 2020). SVHC are subjected to the authorization or restriction process under REACH regardless of their production volume.

One key problem with existing chemical regulations is that there are no effective measures to prevent regrettable substitutions of phased-out chemicals by compounds that pose a similar threat to human and/or environmental health. If one compound is replaced by one or several others, and the production volumes are initially lower, the information requirements for the new chemicals are also lower. Despite the call for a more sustainable material basis for chemical products and processes (Zimmerman et al. 2020), the replacement of phased-out chemicals too often has led to “regrettable substitutions” (Fantke et al. 2015). Substitutions ultimately lead to a higher number of chemicals with a higher degree of structural diversity (Wiesinger et al. 2021) that contribute to the growing gap in testing.

The increasing number and diversity of chemicals in products and their occurrence in the environment have led to the need of dealing with mixtures in chemical risk assessment (Kortenkamp and Faust 2018). There are many examples of elevated mixture toxicity, where exposure to individual chemicals failed to provoke toxicity but in combination, effects are detectable and predictable by established mixture toxicity concepts (Neale et al. 2020; Silva et al. 2002; Walter et al. 2002). While mixture effects have been recognized by the EU (European Commission 2013), chemicals are regulated individually. In line with the precautionary principle, regulatory approaches need to evolve from considering single chemicals to assuring safe levels of chemical mixtures.

During the past decades, there have been only incremental improvements of existing test methods used in regulatory hazard and risk assessments, despite huge gains in the mechanistic understanding of toxicity pathways from scientific studies offering alternative methods to animal testing. As a consequence, basic experimental PBT information was missing for > 98% of a large set of 95’000 organic chemicals on the US and EU markets before REACH (Strempel et al. 2012). Specifically, experimental data on P, B, and acute toxicity were available for only 0.17, 0.78, and 1.73% of these chemicals, respectively. Only for 0.19% of the chemicals, experimental chronic toxicity data were available (Strempel et al. 2012), and strikingly, only 0.07% of these chemicals had a complete set of experimental data on P, B, and T (Strempel et al. 2012). Ten years later, > 40% of REACH-registered substances still had insufficient experimental data for even a crude screening for PMT properties and an alarmingly low 2.2% of > 14,000 chemicals had information on their environmental half-lives (Arp and Hale 2022). This dearth of data does not even include the recognized need for the inclusion of transformation products in risk assessment (Escher and Fenner 2011).

Given this situation, we must ask: what information is really needed, and how can we rapidly obtain this information for substantially more chemicals and their relevant mixtures? We submit that, for the bulk of chemicals currently traded or under development, one has to move away from the idea of highly thorough assessments for a small number of chemicals and instead adopt methods that make it possible to obtain robust information for the comparative assessment of many more chemicals used in commerce, including as mixtures.

The EU has set clear goals with its “Chemicals Strategy for Sustainability towards a non-toxic environment” (CSS) (European Commission 2020). Cornerstones of the CCS include “one substance – one assessment” for improving the efficiency, effectiveness, coherence and transparency safety assessments of chemicals across all relevant legislation (van Dijk et al. 2021). In addition, the CSS recognizes that chemical mixtures must be considered to better protect human health and the environment.

Despite ongoing revisions of risk assessment methods, they continue to rely mainly on conventional in vivo methods for toxicity testing. The US National Academy of Science proposed a paradigm change in risk assessment (U.S. NAS 2017) with a more modern approach to hazard assessment that adopts new approach methodologies (NAMs) and, in particular, high-throughput screening (HTS) techniques (Krewski et al. 2020; NRC 2007). In vitro assays were initially developed to replace animal testing in drug development and cosmetics testing. But with the advances of pathway-based toxicology, it has since been argued that environmental and human health risk assessment could be completely built on HTS (Krewski et al. 2020). For example, HTS results for single chemicals in the Tox21 and ToxCast Programs are made publicly available (Collins et al. 2008; Richard et al. 2021) providing HTS databases supporting health research by identifying putative mechanistic associations and toxicity pathways (Auerbach et al. 2016). These pathways are then linked to adverse health effects and used to prioritize chemicals for in vivo testing (Kassotis and Stapleton 2019). As a result, the US Environmental Protection Agency has committed to HTS techniques for hazard assessment with the decision to phase out vertebrate animal testing by 2035 (U.S. EPA 2021; U.S. EPA 2022).

While REACH is currently under revision, there are fears that revisions would lead to a sharp increase in animal testing if additional testing requirements were mandated without the adoption of NAMs (Zainzinger 2022). In REACH, the so-called integrated testing strategies (ITS) (Rovida et al. 2015) could theoretically allow modern HTS techniques to be used in risk assessment. But their applications have been limited (Sobanska et al. 2014). There have been many debates about the limitations of in vitro assay systems and the lack of proper accounting for in vitro toxicokinetic testing (Blaauboer 2015). This argument overlooks scientific progress rooting HTS assays in adverse outcome pathways (Burden et al. 2015) and unravelling the biokinetics of in vitro assays (Fischer et al. 2018; Proença et al. 2021) that strengthen the scientific basis for the use of HTS assays in risk assessment.

With the unprecedented progress in the development of HTS methods for toxicity assessment, it is time to rethink their application in risk assessment (Birnbaum et al. 2016; Luijten et al. 2020). To date, efforts have been focused on the refinement, reduction, and/or replacement of animal testing, which requires extrapolation of HTS-to*-*in vivo effects (Bell et al. 2018; Wetmore 2015). However, a revision of PBT assessment must go beyond a direct replacement of in vivo methods and instead, develop alternative views on what constitutes a toxicological hazard and how it can be measured. Such approaches must consider the complexity of mixtures and biological relevance while keeping pace with chemical innovation and testing throughput.

This paper outlines the potential of in vitro bioassays as an HT approach for hazard assessment with a focus on toxicological endpoints currently required by REACH plus additional endpoints that are appropriate for next-generation risk assessment. In an earlier communication in this journal, Ball and colleagues (2022) explored how individual tests in REACH can be replaced by NAMs. Here, we build on this work to propose a hazard assessment that considerably streamlines testing requirements while simultaneously accounting for the demand to give PMT chemicals equal weight as PBT chemicals (Hale et al. 2020). Specifically, we propose to develop a measure of total pollution potential based on the two major hazard indicators of persistence and toxicity, and we link these two indicators in an integrated and quantitative approach that facilitates a direct comparison. This would introduce a common “currency” of pollution, facilitating implementation across chemical classes, regulatory agencies, and governments. We suggest that this could be accomplished by testing new chemicals with HTS bioassays, before and after performing one or several (bio-) degradation experiments.

### Status quo of PBT assessment

The identification of persistent, bioaccumulative and toxic (PBT) or very persistent, very bioaccumulative (vPvB) chemicals represents an essential element of REACH (ECHA 2017b) and the Stockholm convention on persistent organic pollutants. PBT assessment under REACH proceeds in two steps, including initial screening based on model predictions and simple screening experiments followed by a definitive assessment. The screening PBT criteria allow computational methods whereas all definitive criteria require that experiments be conducted with whole animals and (fresh) environmental samples.

A classification of a chemical as PBT has significant consequences and may result in a ban (“restriction”) or limited (“authorization”) use. PBT assessment may result in a vicious cycle: if a given chemical is restricted, this will likely, in turn, trigger the development of substitutes. These substitution products may potentially be equally or more harmful than the corresponding existing chemical. But it may take years to reveal if a substitution is safer than its predecessor given the slow, single chemical approach to PBT assessment and hazard classification. In practice, PBT assessment, as well as the more recently promoted PMT assessment, remain limited by a lack of sufficiently high-quality experimental data (Arp and Hale 2022; Strempel et al. 2012).

In persistence assessment, tests for “ready biodegradability” (OECD 301A to 301F (OECD 2002a)) or “inherent degradability” (OECD 302A-F (OECD 2009)) are performed. If degradation in these screening tests does not meet the “degradability” criteria, simulation tests must be performed for a definitive persistence assessment including, for example, tests in soil (OECD 307) (OECD 2002b), sediment–water (OECD 308 (OECD 2002c)), or surface water (OECD 309 (OECD 2002d)). The latter are time-consuming, costly, and need to be performed with radioactively labelled test materials, which additionally complicates and delays their application. Diverse strategies for improvement of persistence assessment have been explored (Whale et al. 2021). Most focus on improving existing methods and or increasing the ability to assess difficult-to-test chemicals (e.g., chemicals with very low solubility).

Bioaccumulation assessment typically relies on the determination of bioconcentration factors (BCF) in fish. Despite major technical improvements in BCF testing (OECD 1996), allowing for dietary exposure (OECD 2012), it is questionable whether the BCF is an appropriate parameter because it relies on aqueous exposure. The route of exposure is not ideal for very hydrophobic chemicals, and this is the main chemical class that are more likely to be bioaccumulative. Alternative measures such as the bioaccumulation factor (uptake via any route) and the biomagnification factor (uptake via food only) would be better suited for hydrophobic chemicals, but they unfortunately continue to rely on animal tests. While integrated testing strategies have been proposed for bioaccumulation assessment (Lombardo et al. 2014), REACH dossiers overwhelmingly contain BCF tests, many of which, for the previously stated reasons, are likely to be flawed, especially for hydrophobic chemicals (Glüge et al. 2022). Furthermore, bioconcentration is a component of toxicity assessment, given that higher bioconcentration factors lead to increased internal concentrations and hence, higher toxicity of a compound.

Mobility (M) is a new criterion, proposed for the hazard classification of CLP, that has yet to be implemented in REACH (Hale et al. 2020; Reemtsma et al. 2016). Current debate centers around use in conjunction with P (then vPvM) or with P and T (then PMT). Currently, several PMT substances have been placed directly in Annex XIV, the list of substances for authorization under REACH, by invoking Article 57(f) of REACH, which allows the inclusion of “substances which give rise to an equivalent level of concern” (EP&EC 2006). If PBT assessment was to be complemented with PMT assessment, it can be foreseen that a large majority of chemicals would either be B or M, largely reducing the assessment to P and T. This is because the current screening threshold for B is an octanol–water partition constant above log *K*_ow_ of 4.5 (ECHA 2017a) and current suggestions for M thresholds are an organic carbon–water partition constant below log *K*_oc_ of 3.0 (for M) and 2.0 (for vM) (Arp and Hale 2022). Since log *K*_ow_ and log *K*_oc_ are rather similar for neutral organics, B and M thresholds are close to overlapping. For instance, of a total of 13,405 unique REACH-registered organic substances (including 445 transformation products thereof) scrutinized in terms of PMT properties by Arp and Hale (2022), there were only 3464 compounds (25.8%) that were not judged M, vM or potentially M. Yet, of those 3464 compounds, two third (i.e., 2291 compounds) had an experimental or estimated log *K*_ow_ or log *K*_oc_ (pH 7) of > 4.5 and would thus be considered bioaccumulative. This leaves a mere 1165 compounds (8.7%) of all REACH registered organic substances that are definitely not M or B according to current criteria.

Environmental toxicity for PBT assessment relies on acute or chronic toxicity testing for aquatic species (ECHA 2017a) and if any measured toxicity exceeds the defined thresholds, the chemical is considered T. Chemicals are considered toxic to human health if classified in GHS category 1A/B for specific target organ toxicity or if they trigger carcinogenicity, mutagenicity and reproduction toxicity (CMR). Endocrine disrupting chemicals (EDC) can also be classified as T, provided they are proven to be “of equivalent concern” (Kassotis et al. 2020). As above, REACH Article 57(a–c) offers the opportunity to directly include chemicals of the GHS category CMR into the authorization list (Annex XIV).

Thresholds for all three criteria (P, B and T) need to be exceeded for a chemical to be classified as a PBT chemical. In Europe, this classification has important consequences as PBT chemicals must undergo a full risk assessment and can be set on the candidate list of SVHCs, which may ultimately lead to their restriction or the need for limited use authorization.

### The potential of new approach methodologies (NAMs)

NAMs comprise all alternative (e.g., tests performed in ≤ 5-day zebrafish or invertebrates such as *Daphnia*) and non-animal (e.g., in silico and in vitro) testing approaches that can inform chemical hazard and risk assessment (U.S. EPA 2021; U.S. EPA 2022). NAMs can be applied in different contexts, for example as replacement of in vivo methods, for read across, or as part of more complex assessment schemes such as IATA (integrated approaches for testing and assessment). NAMs typically also have the capacity to speed up testing (e.g., to produce data for large numbers of chemicals) (Krewski et al. 2020). In practice, in vitro assays have been applied for the risk assessment of individual chemicals (Hatherell et al. 2020; Mone et al. 2020) and mixtures (Bopp et al. 2018; Bopp et al. 2019; Drakvik et al. 2020; Fang et al. 2020).

The Globally Harmonized System of Classification and Labelling (GHS) of chemicals (United Nations 2019) is widely accepted and has been implemented in many national legislations, among them the EU regulation for Classification, Labelling and Packaging (CLP) (EP&EC 2008). The GHS currently relies almost entirely on animal testing (Zainzinger 2022). Beginning in 1994, proposals were made for the adoption of in vitro bioassays for classification and labelling (Seibert et al. 1994). Today, within the GHS, non-animal methods are mainly applied for skin sensitization testing (Ezendam et al. 2016; Hoffmann et al. 2018).

In discussing the potential application of NAMs to PBT assessment, we propose an initial focus on experimental NAMs, including in vitro bioassays and alternative tests (e.g., early life stage zebrafish tests). If the PBT assessment paradigm was shifted from in vivo to in vitro and alternative test methods, then the targets of in silico methods would also need to be adopted to predict in vitro effects. The latter will be possible once sufficient high-quality data are available.

## Proposed new paradigm in PBT/PMT assessment

### Persistence and toxicity as sole hazard indicators

**Fig. 1:**
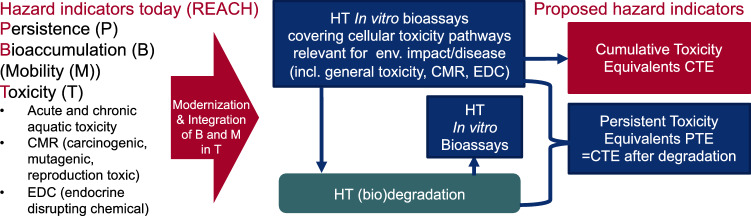
Proposed shift from the traditional PBT indicators implemented in REACH today to our vision of modern high throughput screening indicators that integrate B and M in T and have a joint measure of P and T: cumulative toxicity equivalents (CTE) and persistent toxicity equivalents (PTE). *HT*  high throughput, *CMR* carcinogenic, mutagenic, reproduction toxic, *EDC* endocrine disrupting compounds

Combining the current state of PBT assessment, the need to integrate PMT, and the promise of NAMs, a straightforward solution to making hazard assessment more efficient and effective emerges: Instead of measuring P, B (or M) and T indicators independently with disjointed techniques, we suggest the use of one combined HT method to measure T, before and after simulated degradation, covering both biotic and abiotic degradation processes. The total cumulative toxicity equivalents (CTE) indicator refers to the toxic effects measured directly in a sample (single chemicals, substitution products, or mixtures) and PTE is the equivalent measure of CTE following the degradation of the sample (Fig. 1). For chemicals that do not form toxic transformation products, the reduction in toxicity would directly relate to the degradation of the chemical. Some chemicals may form toxic transformation products following environmental-mediated compound degradation (Escher and Fenner 2011). Such chemicals will also be captured by the proposed approach.

The CTE (Fig. 1) is derived from in vitro and alternative bioassays that cover different environmental and human health-relevant endpoints.

Panels of HTS assays should be selected to align with existing environmental toxicity endpoints, including acute and chronic aquatic toxicity, and the human health hazard triggers of acute toxicity, CMR, and ED and specific organ toxicity (e.g., liver toxicity). The CTE approach also enables simple integration of new organ toxicity measures that are not yet considered in current regulations. These include endpoints where there is emerging societal concern, new scientific evidence, and/or when appropriate in vitro assays become available (e.g., developmental neurotoxicity).

To bring persistence into the equation, any evaluated sample should be subjected to one or more environmental (bio-) transformation processes including biodegradation and abiotic degradation (e.g., hydrolysis, oxidation, reduction, photolysis). The resulting extract can then be re-tested in the HT bioassays to quantify remaining toxicity, termed persistent toxicity equivalents (PTE).

In REACH, any type of degradation is considered in P assessment. However, in practice, the focus is on microbial biodegradation. As it is the most important degradation process for the majority of chemicals, it represents a key biodegradation component within the PTE indicator. Methods for abiotic degradation may be considered as well. HT methods for abiotic degradation are already available (Huchthausen et al. 2022) and may be applied in conjunction with HT biotic degradation to approximate the complete range of degradation processes.

According to current testing paradigms, there is a substantial lack of cost-efficient (bio)degradation systems that go beyond the series of OECD guidelines for testing of “ready biodegradability” (OECD 301) and “inherent biodegradability” (OECD 302). These tests present a challenge for chemical development because approximately 50% of the REACH chemicals for which those data are reported (Table S1 in (Arp and Hale 2022)) failed these tests. If chemicals fail the tests for ready and inherent biodegradability, cumbersome and expensive simulation studies are advised that require ^14^C-labelled compounds (e.g., OECD 307–309). With the proposed CTE/PTE approach, ^14^C-labeling and chemical analysis become obsolete because only the mixture effects of a parent compound and its associated transformation products in the degraded samples will be quantified, yet their chemical composition will not need to be resolved. This allows for more cost-efficient testing in a tiered strategy. These new methods, provided they meet the expectations in several case studies, can then be developed into standards as, for example, OECD testing guidelines.

### CTE: a range of toxicity endpoints

In vitro bioassays have yet to be used as formal hazard indicators in PBT assessment. However, given the concordance between the critical cellular concentrations in aquatic animals and cell systems (Escher et al. 2019), in vitro and alternative bioassays also have the potential to be used to assess aquatic hazard. The acute and chronic toxicity endpoints measured for algae, daphnids and fish, which are required in PBT assessment, could be directly replaced by appropriate cellular toxicity systems (Fischer et al. 2019; Schug et al. 2020; Tanneberger et al. 2013), high throughput algae (Glauch and Escher 2020), or invertebrate testing (Castro et al. 2019), or the fish embryo toxicity test (Scholz et al. 2014; Teixido et al. 2019). Cytotoxicity can be used as proxy for acute toxicity in humans and the environment (Lee et al. 2021).

For environmental hazards (Sakamuru et al. 2020) and the health hazard triggers of CMR (carcinogenic, mutagenic and reproduction toxic) and EDC (endocrine disrupting chemical), a suite of cell-based bioassays is available (Kleinstreuer et al. 2015; Shah et al. 2011; Smith et al. 2016; Toporova and Balaguer 2020). Thousands of chemicals have already been screened with HT bioassays in the Tox21/ToxCast initiatives (Betts 2013; Krewski et al. 2020), which allows for the use of considerable existing information in CTE/PTE determination. We propose to stay close to the demands of the existing T endpoints in REACH’s PBT assessment, and to gradually expand the CTE/PTE approach to a much broader range of toxicity pathways and organ toxicities with the future development of suitable assays.

As the Tox21/ToxCast assays only cover early events in the cellular toxicity pathway and lack coverage for more complex endpoints, it will be necessary to work with several bioassays in parallel. More work is needed to determine how many and which bioassays will be necessary to provide a comprehensive assessment. Since the research area of adverse outcome pathways and bioassays is rapidly evolving, we anticipate that there will be abundant in vitro assays available for inclusion in the CTE/PTE testing scheme in the near future, including adaptive stress responses and developmental neurotoxicity. For example, unlike pesticides and pharmaceuticals, industrial chemicals and consumer products are not designed to have one dominant and highly specific/selective mode of toxic action but may cause many different effects that can be characterized by diverse molecular initiating events followed by often convergent key events and adaptive stress responses. Hence, adaptive stress responses have the potential to complement key molecular initiating events in a more integrative testing (Simmons et al. 2009).

Effects on reproduction, development and developmental neurotoxicity (DNT) are among the adverse outcomes considered most crucial to human health (Grandjean and Landrigan 2006). However, existing health hazard triggers do not incorporate important organ toxicity hazards such as DNT, which has come into focus due to the rising incidence of neurological disorders such as autism (Rossignol et al. 2014) and the potential association with chemical exposure (Bennett et al. 2016). A primary reason why DNT has not been considered in hazard assessment is that established in vivo approaches (OECD 426 for DNT) are extremely costly, ethically questionable, and there are concerns regarding their predictivity (Masjosthusmann 2020). Recent development of screening approaches using cellular and zebrafish embryo assays now enable the inclusion of DNT endpoints suitable for comparative assessment of chemicals (Behl et al. 2019; Blum et al. 2023).

### PTE: HT degradation coupled to HT toxicity screening

The CTE/PTE approach automatically integrates potentially toxic transformation products into hazard assessment. After degradation, the toxicity/effects are typically reduced in parallel with the decrease of the parent compound, but if potent transformation products are formed, they contribute to the mixture effect that constitutes the PTE. Since transformation kinetics and pathways might vary as a function of environmental conditions, both for biotic and abiotic degradation processes, the biggest challenge of the approach is the establishment and evaluation of HT (bio-) degradation assay set-ups to achieve environmentally realistic attenuation of organic chemicals in an HT environment. Since it is a screening approach, the goal is to obtain a realistic picture of the transformation potential of a chemical and to capture the formation of any toxic and persistent transformation product (Boxall et al. 2004; Escher and Fenner 2011). In an HT set-up, not only biotic but also abiotic degradability could be evaluated with a focus on major, environmentally relevant abiotic processes, i.e., hydrolysis, abiotic oxidation and direct and indirect photodegradation.

As compared to current biodegradation testing in a hazard assessment context, the proposed new paradigm includes the assessment of degradation and formation of transformation products that are not tracked with chemical analysis. No degradation rate constants of the parent chemical will be derived, but rather the reaction mixture will be extracted and then subjected to repeated bioassay testing. This could be done repeatedly over multiple time points to obtain a “toxicity reduction rate” or at a final time point to obtain a “toxicity reduction ratio”.

As part of a hazard-based approach, this is sufficient to assure that degradation of the chemical or mixtures occurs and that no toxic transformation products are formed during degradation. Of course, if toxicity persists, it cannot be known whether it is due to parent stability or formation of toxic transformation products. However, for the assessment of “persistent toxicity”, it does not matter which scenario applies as either is problematic. The PTE assessment would yield sufficient information at this stage to make a use determination.

To ensure consistency with previously generated degradation data and persistence assessments, the HT biodegradation assays should have a similar transformation efficiency as the traditional biodegradation simulation tests (OECD307-309). Ideally, they would also include microbial communities from diverse environments to appropriately represent the diversity of degrading bacteria. For ready biodegradability tests, first HT test systems covering a range of conditions and bacterial communities from different environments to yield a degradation probability have been established, demonstrating the feasibility of HT degradation systems (Brillet et al. 2016; Martin et al. 2017). At the moment, the need for a fast, e.g., colorimetric read-out for degradation limits wider application. This issue will be overcome by PTE because the measured toxicity is the readout.

To benchmark the performance of new HT biodegradation assays against existing biodegradation simulation tests, a set of benchmark chemicals could be defined that include data-rich chemicals covering the relevant range of persistence behavior (Honti et al. 2018, 2016; Seller et al. 2021). These could be used to develop HT test systems to ensure that they achieve similar degrees of degradation as existing simulation biodegradation tests, but ideally in a shorter time frame. To achieve the latter, one would need to move beyond strictly mirroring conditions of the OECD test guidelines and instead, maximize degradation capacity by, for example, using higher bacterial densities or increasing nutrient status and oxygenation levels.

It is conceivable that HT biodegradation assays could eventually be developed toward assembled communities of many well-characterized bacterial strains or microbes enriched from different environments, taking full advantage of robotic systems to assemble, test and optimize them. This could potentially provide more stable and reproducible test systems as the bacterial inocula, once optimized, could be prepared in large batches, frozen and made available to CTE/PTE screeners. Such consensus cell lines or assembled inocula would represent a breakthrough in biodegradation testing because experiments could then be repeated over time in the same laboratory and in many laboratories worldwide, yielding the same results (within a range of uncertainty and variability). This would allow for the compilation of a large and robust databases of in vitro biodegradation responses which, in turn, could be used for the development of in silico biodegradation models, an effort that is currently hindered by the small size of available data sets from experimental biodegradation studies.

## *Experimental challenges of *in vitro* testing after biodegradation*

The practical challenge for bioassay application is to ensure that they are compatible with testing samples after performance of the HT biodegradation assay. Here, we can rely on the experiences of application of in vitro bioassays for water quality monitoring and biomonitoring. Complex mixtures in water and other matrices are typically extracted with solid-phase extraction (Escher et al. 2021) and many studies have evaluated the SPE recoveries for highly diverse chemicals, which were found to be satisfactory for bioactive mixtures (Neale et al. 2018) and mobile chemicals (Stalter et al. 2016).

### CTE/PTE suitable for comparative assessment of replacement products

The regrettable substitution of chemicals is most often caused by inadequate and slow assessment of replacements. A well-known example of this phenomenon was the ban of bisphenol A (BPA) (Fouyet et al. 2021). By the time BPA was banned in 2020, numerous substitutes had already entered the market and were detected in a range of human and environmental samples (Bennett et al. 2016; Ji et al. 2021). As registration of new substances is only required above 1 metric ton per year, replacement products may sneak into the market with insufficient hazard and risk assessment, when one chemical is substituted by many different replacement chemicals, generated in lower quantities.

Upon closer inspection, replacement chemicals often carry undesirable properties very similar to the phased-out chemicals. Many substitutions are structurally related and/or cause similar toxicity (den Braver-Sewradj et al. 2020; Fantke et al. 2015; Thoene et al. 2020). For example, plastics contain a range of regulated and non-regulated brominated flame retardants with bromine mass balances indicating that the fraction of non-regulated brominated flame retardants is high (Hennebert 2021). Public perception might favor unknown relative to known risks. For instance, consumers who opted for “BPA-free” products were inadvertently selecting for alternative products containing hazardous BPA analogues (Scherer et al. 2014). When informed about alternative products and their hazards, consumers still often chose the alternative even there is less known about their hazard (Scherer et al. 2014).

Maertens et al. (2021) argued that regrettable substitutions for BPA have occurred although extensive knowledge exists on the adverse effects of the substitution products (Keminer et al. 2020; Liang et al. 2020; Pang et al. 2019). However, because these studies were academic studies, often focused on molecular or cellular alterations and not performed with in vivo assays accepted in regulatory risk assessment, they were often not considered within regulatory risk assessments.

Replacement of C8-PFAS to shorter chain homologues resulted in the desired lower potential of bioaccumulation, but similar persistence and a significantly increased mobility (Brendel et al. 2018). In contrast, the structural similarity of the replacement compound hexafluoropropylene oxide dimer acid (HFPO-DA) in comparison with the regulated perfluorooctanoic acid (PFOA) led to similar persistence and long-range transport potential (Joerss et al. 2020). Consequently, both alternatives should be considered as regrettable. A similar example is Bisphenol S (BPS). BPS has a lower environmental toxicity than BPA but is more resistant to environmental degradation and causes more pronounced changes in the host associated microbiome (Catron et al. 2019). It, therefore, does not solve the problem posed by BPA.

It will take years to assess and regulate unsuitable substitution products one-by-one. CTE/PTE hazard indicators would remediate that situation because they enable a direct comparative assessment of a chemical and its substitution products (S1, S2, etc.), while considering bioactivation and detoxification events. This direct comparison removes the need for in vivo data on the substitute. In the suggested approach, CTE and PTE of chemicals and their substitution products can be directly compared and only substitutions with better screening outcomes would be considered as potentially suitable replacements and undergo a full risk assessment. This strategy could therefore be used for the targeted development of benign-by-design substitution products, a tenant of the green chemistry movement.

**Fig. 2:**
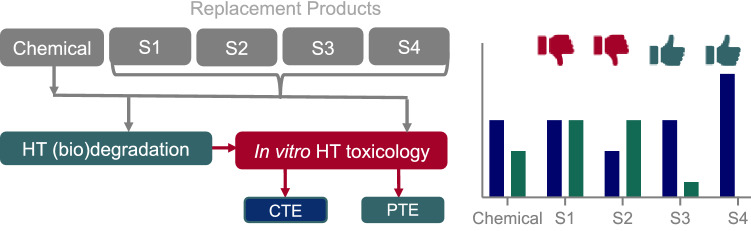
Theoretical application of CTE/PTE hazard indicators for comparative assessment of a chemical and its substitution products

The strategy to avoid regrettable substitutions is illustrated in Fig. 2. As an example, the CTE of the chemical of interest for a certain bioassay is reduced by 40% after degradation. Any substitution chemical should have lower CTE and PTE values. In the example, the substitution chemical S1 has the same CTE but is persistent, so CTE = PTE, and PTE is higher for S1 than the original chemical, which makes S1 unfit as replacement.

Although S2 has an apparent lower toxicity (CTE), it forms toxic transformation products, so PTE > CTE, which is not acceptable, despite CTE is lower for S2 than the original chemical. The inclusion of the toxicity of transformation products demonstrates one considerable advantage of including the PTE indicator. If only primary degradation and toxicity of a chemical is considered, the formation of persistent and potentially toxic transformation products could be overlooked. In this example, only S3 and S4 could be considered as suitable replacement products. S3 has similar CTE relative to the original chemical but lower PTE, while S4 seems initially to be much worse with a higher CTE but is fully degradable, leaving no trace of toxicity. Hence, educated choices can be made, depending on use, for a substitution product with higher CTE but lower PTE because this would mean that the substitution product would be less problematic than the original substance.

### Incorporating mixtures into hazard assessment

Numerous chemicals have been detected in parallel in the environment and in humans (Escher et al. 2020). We submit that any method that is applicable for testing of chemicals should also be applicable for use in biomonitoring and to test environmental mixtures. This concept applies to undefined substances of unknown composition such the UVCB (**U**nknown or **V**ariable composition, **C**omplex reaction products or **B**iological materials), which pose a major challenge in risk assessment (ECHA 2017c; Lai et al. 2022). Any novel approach to hazard assessment must be versatile enough to be applicable for the assessment of mixtures and UVCB to meet the demands of the CSS (European Commission 2020). Not only can the CTE/PTE indicators be measured with single chemicals and used to rank the hazard of chemicals, they can also be measured for UVCBs and other intentional and unintentional mixtures, for example those found in consumer products.

Consumer products are made up of many different chemicals. It has been estimated that people may place over 500 chemicals on their skin through cosmetics use alone (www.science.org.au/curious/people-medicine/chemistry-cosmetics) that typically contain emulsifiers, preservatives, thickeners, emollients, dyes, fragrance materials and pH stabilizers, preservatives, UV-filters, antioxidants, and impurities from the production process (Tang et al. 2021). While mixture exposure is evident, hazard potentials remain unclear for such intentional mixtures from products and unintentional mixtures from the use of many products because the risk assessment is performed at the level of individual chemicals, not on the level of the consumer product.

Since CTE/PTE measure the effect before and after degradation, it is amenable to mixtures in products, including first use and recycled consumer products. Recycled products were shown to contain many more chemical features than virgin products in analytical suspect screening, but little is known about the comparative hazard of virgin versus recycled products (Lowe et al. 2021).

### Benefits of the novel hazard indicators CTE/PTE for chemicals regulation and society

A sustainable and circular use of chemicals is a major goal of decision makers in the EU and beyond. This goal is in contrast with the open uses of many chemicals. Open uses are generally dispersive and therefore not circular (and can never be). Therefore, the way in which chemical products are used has to fundamentally change to establish a circular economy (Kümmerer et al. 2020) that is adequately protective of human and environmental health.

To reach the goal of a sustainable use of chemicals, decision-makers need to address many existing societal and regulatory challenges. These challenges include a very complex, expert-led regulatory infrastructure focused on the authorization of single chemical substances; a lack of inclusion of civil society groups in regulatory processes at the EU level and in member states; and consumer interests that can only be met through the use of synthetic chemicals (e.g., cosmetics can be stored over a long time in a warm bathroom) (Hüesker and Lepenies 2022).

The new CTE/PTE indicators introduced here provide an opportunity to address these challenges by (1) increasing the transparency of PBT assessment and basing it on experimental data rather than simulated models, (2) supporting the application of the precautionary principle, and 3) helping to initiate responsible innovation processes.

### CTE/PTE can support the application of the precautionary principle

The precautionary principle is a major guiding principle for decision-makers faced with emerging risks, scientific uncertainty, and public concerns (Aven 2011). The precautionary principle has provided an important guidance for situations of uncertainty, where decisions, e.g., about chemicals’ marketing or regulation, must often be taken in situations of lacking data (Klinke and Renn 2001). In situations where possible negative impacts are still uncertain, the precautionary principle allows decision-makers to impose regulatory actions when there are reasonable grounds for concern. Clear guidelines for implementing the precautionary principle are lacking and the principle has been criticized for hindering technological innovation and for neglecting countervailing risks (Lofstedt 2014). In this situation, CTE/PTE can aid the application of the precautionary principle in chemicals management in a systematic way.

As a fast and simple screening tool, CTE/PTE would permit testing of any chemical/substitution product prior to registration under REACH as a precautionary measure or directly after registration. If, at that point, rapid in vitro screening of the CTE/PTE indicators suggests that there is the potential of a new entity or a replacement product to be hazardous, it should not be marketed without firm evidence that the in vitro results do not translate to in vivo hazard. Beyond that, industry could preemptively use the CTE/PTE indicators during the research and development phase to avoid investing development time and costs into environmentally problematic chemicals. Based on its potential for simplified testing, CTE/PTE would answer current calls for taking multiple use cycles into account when assessing hazards and risks to reach “sustainable circularity” (Wang and Hellweg 2021).

### CTE/PTE can increase the transparency of chemical safety assessment

Risk management on the basis of hazard characterization of substances and mixtures could initiate a review of current legal pathways to justify risk management. Currently, PBT assessment represents a crucial first step of the overall chemical safety assessment where a chemical is declared “not PBT” even if assessed only by screening criteria using predicted data or very simple screening tests. If it is deemed to be PBT, it will not undergo further chemical safety evaluation unless it fulfills the criteria for classification under CLP (ECHA 2017b). PBT assessment relies on data of variable quality for an important decision. In most cases, transformation products are not considered in these assessments. Implementation of the CTE/PTE indicators would assure that the decision is based on comparable experimental data that facilitate inclusion of transformation products into the evaluation, thus allowing for transparent and data-driven decisions.

### CTE/PTE can help initiate and sustain responsible innovation

In parallel with developing the technical basis for CTE/PTE, it is pivotal to include relevant stakeholders including regulatory agencies, civil society, and industry to ensure that innovations like CTE/PTE meet societal needs and respond to pressing societal challenges (Owen et al. 2021). To achieve this goal, we propose a transdisciplinary approach in the development of CTE/PTE as novel hazard concept, consisting of the following three aspects. One, conduct focus groups with key societal stakeholders, including industry and civil society organizations, to systematically explore their needs and reaction to the CTE/PTE indicators. Two, organize scenario workshops and case studies based on quantitative data generated by hazard assessments and qualitative data from the focus groups to describe plausible visions of the future and demonstrate alternatives to the ‘‘*business as usual*’’. This will open up the space of options for future deployment of CTE/PTE by stimulating discussion and an exchange of arguments and ideas amongst stakeholders. Three, develop and communicate shared narratives about the value of chemicals testing and pathways for future regulation (Leipold 2021). This inclusive and transdisciplinary strategy allows for the integration of scientific evidence, stakeholder concerns, and the public for more comprehensive hazard and risk assessment and improved regulation of chemicals (Malakar et al. 2022; Von Schomberg 2019). Collectively, this strategy can provide crucial guidance for the implementation of the CTE/PTE concept and key operative and regulatory innovations.

## Conclusion

The proposed approach to base hazard assessment exclusively on in vitro and alternative testing has the potential to modernize hazard assessment as envisioned for twenty-first century toxicology and risk assessment (Krewski et al. 2020). With this paper, we aim to spark a debate. We can rely on existing in vitro and alternative assays to begin, but we still need to develop coherent and robust batteries of bioassays to measure the hazard indicators CTE and PTE and formally adopt them in testing guidelines.

The novel indicators CTE/PTE provide hazard assessment with a new, comprehensive perspective. To leverage its high innovation potential, this requires a new paradigm within chemical regulation. The CTE/PTE approach must meet legal requirements and fit the EU zero pollution ambition for a toxic-free environment. To achieve this goal, important elements remain to be implemented and critically evaluated, such as robustness, repeatability, quality assurance/quality control, consistency with present PBT outcomes, blind spots and guidance document development.

This paper is our attempt to accelerate the implementation process, encourage researchers to start activities, and engage stakeholders from the regulatory, industrial, and public sectors to discuss their needs and identify key areas that require additional innovation. In the end, CTE and PTE might not be realized exactly as we envisioned it here. But we hope that our ideas serve as a nucleus for an accelerated implementation of NAMs in hazard assessment to modernize PBT assessment. While we aim to initiate this strategy in European Union-funded research and innovation programs, if proven useful, we propose that this regulatory concept be applied to ultimately improve protection measures for human health and the environment.

CTE and PTE can be used in prospective hazard assessment for the development of substitution chemicals or new products as outlined above. CTE might also have potential for application in the GHS and CLP similar to what has recently been outlined in this journal by Ball and colleagues (2022). The CLP has eight health hazard categories and for each, suitable in vitro and alternative assays are needed. With the development of adverse outcome pathways as structuring principles in mechanistic toxicology, it is possible to match appropriate in vitro assays to adverse outcomes of interest (Carusi et al. 2018).

While we emphasized in vitro and alternative methods in this article, we want to stress that they can be complemented by in silico approaches in the future. This will further increase the speed of assessments while simultaneously decreasing costs (Muratov et al. 2020). Considering the increasing power of in silico methods, these approaches can be also used to complement in vitro measurements to prioritize compounds for testing (Abdelaziz et al. 2016). As CTE/PTE is a new approach, the experimental database still needs to be expanded before in silico prediction methods, which are also subsumed under the term NAMs, can be develop and productively applied. Predictive methods, including AI-supported and machine learning approaches (Fantke et al. 2021; Wu et al. 2021) have a great potential to further advance the use of NAMs in hazard and risk assessment.

The unprecedented advantage of the proposed approach is that the same indicators, CTE and PTE can be used for both prospective hazard assessment and retrospective assessment by incorporating these tools in (bio)monitoring. This will provide a universal parameter for chemical pollution that can be used to monitor chemical pollution and can be integrated into a global chemical risk assessment strategy. CTE and PTE would be a common currency across regulatory silos and enable the assessment of past pollution and guide future development of safer chemicals.

## Data Availability

This conceptual paper did not use any data and did not perform formal data analysis.

## Abbreviations

3Rs: Refine, Reduce, or Replace animal testing
BAF: Bioaccumulation Factor
BCF: Bioconcentration Factor
CLP: EU Regulation for Classification, Labelling and Packaging
CMR: Carcinogen, Mutagen, Reproduction toxicant
CSS: Chemicals Strategy for Sustainability towards a non-toxic environment
CTE: Cumulative Toxicity Equivalent
DNT: Developmental Neurotoxicity
ED: Endocrine Disruption
EDC: Endocrine Disrupting Chemical
EU: European Union
GHS: Globally Harmonized System of Classification and Labeling
HT: High Throughput
HTS: High Throughput Screening
IATA: Integrated Approaches for Testing and Assessment
ITS: Integrated Testing Strategy
NAM: New Approach Methodology
PBT: Persistent, Bioaccumulative, Toxic
PFAS: Per- and Polyfluoroalkyl Substances
PMT: Persistent, Mobile, Toxic
POP: Persistent Organic Pollutant
PTE: Persistent Toxicity Equivalent
REACH: European Union’s Regulation on the Registration, Evaluation, Authorization and Restriction of Chemicals
SVHC: Substance of Very High Concern
UVCB: Mixture of Unknown or Variable composition, Complex reaction products or Biological materials
vPvB: Very Persistent, very Bioaccumulative
vPvM: Very Persistent, very Mobile
